# Supplementary material for: Rehabilitation Interventions for Post-Acute COVID-19 Syndrome: A Systematic Review
Source: Int J Environ Res Public Health. 2022 Apr 24;19(9):5185. doi: 10.3390/ijerph19095185 (PMC9104923; doi:10.3390/ijerph19095185)
Supplement: Supplementary file 1 [file ijerph-19-05185-s001.zip › ijerph-1670495-supplementary.pdf]

File S1 - PRISMA 2020 Checklist

Section and Topic	Item #	Checklist item	Location where item is reported
**TITLE**			
Title	1	Identify the report as a systematic review.	Title
**ABSTRACT**			
Abstract	2	See the PRISMA 2020 for Abstracts checklist.	Abstract
**INTRODUCTION**			
Rationale	3	Describe the rationale for the review in the context of existing knowledge.	Page 2 Introduction
Objectives	4	Provide an explicit statement of the objective(s) or question(s) the review addresses.	Page 3. Last paragraph of Introduction
**METHODS**			
Eligibility criteria	5	Specify the inclusion and exclusion criteria for the review and how studies were grouped for the syntheses.	Page 3, Materials and Methods
Information sources	6	Specify all databases, registers, websites, organisations, reference lists and other sources searched or consulted to identify studies. Specify the date when each source was last searched or consulted.	Pages 3-4, Search strategy and File S2
Search strategy	7	Present the full search strategies for all databases, registers and websites, including any filters and limits used.	Appendix B
Selection process	8	Specify the methods used to decide whether a study met the inclusion criteria of the review, including how many reviewers screened each record and each report retrieved, whether they worked independently, and if applicable, details of automation tools used in the process.	Page 4 Data collection and analysis
Data collection process	9	Specify the methods used to collect data from reports, including how many reviewers collected data from each report, whether they worked independently, any processes for obtaining or confirming data from study investigators, and if applicable, details of automation tools used in the process.	Page 4 Data extraction and management
Data items	10a	List and define all outcomes for which data were sought. Specify whether all results that were compatible with each outcome domain in each study were sought (e.g. for all measures, time points, analyses), and if not, the methods used to decide which results to collect.	Page 4 Data extraction and management
	10b	List and define all other variables for which data were sought (e.g. participant and intervention characteristics, funding sources). Describe any assumptions made about any missing or unclear information.	Page 4 Data extraction and management
Study risk of bias assessment	11	Specify the methods used to assess risk of bias in the included studies, including details of the tool(s) used, how many reviewers assessed each study and whether they worked independently, and if applicable, details of automation tools used in the process.	Page 4 Data extraction and management
Effect measures	12	Specify for each outcome the effect measure(s) (e.g. risk ratio, mean difference) used in the synthesis or presentation of results.	Page 4 Data extraction and management

File S1 - PRISMA 2020 Checklist

Section and Topic	Item #	Checklist item	Location where item is reported
Synthesis methods	13a	Describe the processes used to decide which studies were eligible for each synthesis (e.g. tabulating the study intervention characteristics and comparing against the planned groups for each synthesis (item #5)).	Pages 4-5 Synthesis
	13b	Describe any methods required to prepare the data for presentation or synthesis, such as handling of missing summary statistics, or data conversions.	Pages 4-5 Synthesis
	13c	Describe any methods used to tabulate or visually display results of individual studies and syntheses.	Pages 4-5 Synthesis
	13d	Describe any methods used to synthesize results and provide a rationale for the choice(s). If meta-analysis was performed, describe the model(s), method(s) to identify the presence and extent of statistical heterogeneity, and software package(s) used.	NA
	13e	Describe any methods used to explore possible causes of heterogeneity among study results (e.g. subgroup analysis, meta-regression).	NA
	13f	Describe any sensitivity analyses conducted to assess robustness of the synthesized results.	NA
Reporting bias assessment	14	Describe any methods used to assess risk of bias due to missing results in a synthesis (arising from reporting biases).	Page 4 Data extraction and management
Certainty assessment	15	Describe any methods used to assess certainty (or confidence) in the body of evidence for an outcome.	NA
**RESULTS**			
Study selection	16a	Describe the results of the search and selection process, from the number of records identified in the search to the number of studies included in the review, ideally using a flow diagram.	Figure 1
	16b	Cite studies that might appear to meet the inclusion criteria, but which were excluded, and explain why they were excluded.	Figure 1
Study characteristics	17	Cite each included study and present its characteristics.	Page 5, Results, Study selection and Table 1
Risk of bias in studies	18	Present assessments of risk of bias for each included study.	Figure 2 and Pages 5-7, methodological quality
Results of individual studies	19	For all outcomes, present, for each study: (a) summary statistics for each group (where appropriate) and (b) an effect estimate and its precision (e.g. confidence/credible interval), ideally using structured tables or plots.	Tables 2 A-B-C
Results of syntheses	20a	For each synthesis, briefly summarise the characteristics and risk of bias among contributing studies.	NA
	20b	Present results of all statistical syntheses conducted. If meta-analysis was done, present for each the summary estimate and its precision (e.g. confidence/credible interval) and measures of statistical heterogeneity. If comparing groups, describe the direction of the effect.	NA
	20c	Present results of all investigations of possible causes of heterogeneity among study results.	NA
	20d	Present results of all sensitivity analyses conducted to assess the robustness of the synthesized results.	NA
Reporting biases	21	Present assessments of risk of bias due to missing results (arising from reporting biases) for each synthesis assessed.	NA

File S1 - PRISMA 2020 Checklist

Section and Topic	Item #	Checklist item	Location where item is reported
Certainty of evidence	22	Present assessments of certainty (or confidence) in the body of evidence for each outcome assessed.	NA
**DISCUSSION**			
Discussion	23a	Provide a general interpretation of the results in the context of other evidence.	Discussion Pages 18-20
	23b	Discuss any limitations of the evidence included in the review.	Discussion Pages 20-21
	23c	Discuss any limitations of the review processes used.	Discussion Pages 20-21
	23d	Discuss implications of the results for practice, policy, and future research.	Discussion Pages 19-21
**OTHER INFORMATION**			
Registration and protocol	24a	Provide registration information for the review, including register name and registration number, or state that the review was not registered.	Abstract and Page 3, Material and Methods
	24b	Indicate where the review protocol can be accessed, or state that a protocol was not prepared.	Abstract and Page 3, Material and Methods
	24c	Describe and explain any amendments to information provided at registration or in the protocol.	NA
Support	25	Describe sources of financial or non-financial support for the review, and the role of the funders or sponsors in the review.	Page 22 - Funding
Competing interests	26	Declare any competing interests of review authors.	Page 22 – Conflicts of Interest
Availability of data, code and other materials	27	Report which of the following are publicly available and where they can be found: template data collection forms; data extracted from included studies; data used for all analyses; analytic code; any other materials used in the review.	Page 22 - Data Availability Statement

From: Page MJ, McKenzie JE, Bossuyt PM, Boutron I, Hoffmann TC, Mulrow CD, et al. The PRISMA 2020 statement: an updated guideline for reporting systematic reviews. BMJ 2021;372:n71. doi: 10.1136/bmj.n71 For more information, visit: http://www.prisma-statement.org/

File S2: Search strings for all databases in the systematic review.

Database searched	Search terms
**Medline, Embase, Cochrane Register of Controlled Trials, CINHAL, Scopus, Prospero, PEDro**	(“long COVID” OR “chronic COVID syndrome” OR “chronic COVID-19” OR “COVID long-hauler” OR “COVID-19 long-hauler” OR “long haul COVID” OR “long hauler COVID” OR “post COVID 19 fatigue” OR “post COVID 19 neurological syndrome” OR “post COVID 19 syndrome” OR “post COVID fatigue” OR “post COVID impairment” OR “post COVID syndrome” OR “post-acute COVID syndrome” OR “post-acute COVID-19” OR “post-acute COVID-19 fatigue” OR “post-acute COVID-19 neurologica syndrome” OR “post-acute sequelae of SARS-CoV-2 infection” OR “post-acute COVID19 syndrome”)<br>**AND**<br>(Rehab* OR “Activities of Daily Living” OR “ADL” OR “Animal Assisted Therapy” OR “Equine Assisted Therapy” OR “Art Therapy” OR Bibliotherapy OR “Correction of Hearing Impairment” OR Lipreading OR “Manual Communication” OR “Dance Therapy” OR “Early Ambulation" OR “Exercise Therapy” OR “Endurance Training” OR “Muscle Stretching Exercises” OR “Plyometric Exercise” OR “Resistance Training” OR “Music Therapy” OR “Occupational Therapy” OR “Recreation Therapy” OR “Language Therapy” OR “Myofunctional Therapy” OR “Speech Therapy” OR “Voice Training” OR “Horticultural Therapy” OR Telerehabilitation OR “Physical Therapy Modalit*” OR “Electric Stimulation Therapy” OR “Extracorporeal Shockwave Therapy” OR Hydrotherapy OR “Musculoskeletal Manipulations” OR “functional status” OR “Self care” OR Habilitation OR “functional readaptation” OR “medical rehabilitation” OR readaption OR readjustment OR resocialization OR “revalidation” OR rejuvenation OR “self report” OR “sensorimotor integration” OR “mirror therapy”)
**International Clinical Trials Registry Platform (WHO)**	“LONG COVID” **AND** “REHABILITATION”

SUPPLEMENTARY

TABLES

Table S1. Characteristics of the experimental interventions and controls

Study	Sample	Intervention: Control	Intervention: Experimental	Frequency (times/wks)	Session Length	Program Duration	Setting	Modality
**Li et al., 2021**	EG: 59<br>CG: 61		Unsupervised home-based exercise programme comprising breathing control and thoracic expansion, aerobic exercise and LMS exercise, delivered via smartphone, and remotely monitored with heart rate telemetry.	3-4	40-60 min	6 wks	Home-based via App	Un-Supervised
		Short educational instructions at baseline		-	-	-	-	-
**Nambi et al., 2022**	EG: 38<br>CG: 38	Supervised 30 minutes of Low intensity aerobic training (40%–60% max HR) in an exercises program including 15 min warm up, 30 min resistance training and 15 min cool down	Supervised 30 minutes of High intensity aerobic training (60% - 80% max HR) in an exercises program including 15 min warm up, 30 min resistance training and 15 min cool down	4	90 min	8 wks	Pt Department	Supervised In presence
**Srinivasan et al., 2021**	EG: 24<br>CG: 24		Pursed lip breathing exercise with bhastrika pranayama	21 (thrice a day)	5 min	6 wks	Home-based	Un-Supervised
		Incentive spirometry		21 (thrice a day)	5-10 reps	6 wks	Home-based	Un-Supervised
**Liu et al., 2020**	EG: 38<br>CG: 38		a) Respiratory muscle training; Cough exercise; Diaphragmatic training; Stretching exercise	2	10 min	6 wks	-	-
			b) Home exercise (pursed-lip breathing and coughing training)	7	30 sets			
		No rehabilitation intervention		-	-	-	-	-
**De Souza et al., 2021**	EG: 104<br>CG: 92		Low-intensity Pulmonary Rehabilitation Program	1	??	6 wks	Home-based	Video-conference
		Only drug intervention		??	??	6 wks	-	-

LMS lower-limb muscle strength; *EG* experimental group; *CG* control group; *wks* weeks; *min* minutes; *HR* heart rate; *reps* repetitions; *HE* Home Exercises.

Table S2. Outcome Measures Used in the Trials Included in the Review (**Primary** and Secondary)

Study	Symptoms		Functional Outcomes			Quality of Life and Independence Outcomes
	Fatigue & Dyspnoea	Mood Disturbances	Pulmonary Function	Muscle Mass & Strength	Functional Exercise Capacity	
**Li et al., 2021**	mMRC	-	FEV1 (L), FVC (L), FEV1/FVC%, MVV (L/min), PEF (L/s)	Static Squat Test at the wall	**6MWT**	SF-12
**Nambi et al., 2022**	-	TSK-11	-	**Handgrip strength**; Cross-sectional areas at MRI of biceps, quadriceps and calf muscles	-	SARQoL
**Srinivasan et al., 2021**	-	-	**FVC, FEV1**	-	-	-
**Liu et al., 2020**		SAS; SDS	**FEV1, FVC, FEV1/FVC ratio, DLCO**		6MWT	SF-36, FIM
**De Souza et al., 2021**	RPE	-	-	-	**STS**, PADL	-

mMRC = modified British Medical Research Council dyspnoea score; *FEV1* = Forced Expiratory Volume in the 1st second; *FVC* = Forced Vital Capacity; *MVV* = Maximal Voluntary Ventilation; *PEF* = Peak expiratory flow; *6MWT* = 6 minutes walking test; *SF-12* Short Form Survey-12; *TSK-11* = Tampa scale of kinesiophobia; *SARQoL* = Sacopenia and Quality of Life; *RPE* = Rating of Perceived Exertion by the 10-points Borg Scale; *STS* = 30 seconds Sit to Stand Test; *PADL* = Physical Activity in Daily Live; *SAS* = Self Rating Anxiety Rate; *SDS* = Self Rating Depression Scale; *DLCO* = diffusing lung capacity for carbon monoxide; *SF-36* = Short Form Survey-36; *FIM* = Functional Independence Measure.
